# Glutathione Peroxidase Level in Patients with Vitiligo: A Meta-Analysis

**DOI:** 10.1155/2016/3029810

**Published:** 2016-04-27

**Authors:** Bi-huan Xiao, Meihui Shi, Hongqiang Chen, Shaoshan Cui, Yan Wu, Xing-Hua Gao, Hong-Duo Chen

**Affiliations:** ^1^Department of Dermatology, No. 1 Hospital of China Medical University, Shenyang 110001, China; ^2^Department of General Surgery, Shandong Qianfoshan Hospital, Jinan 250014, China; ^3^Department of Dermatology, The First Affiliated Hospital of Dalian Medical University, Dalian 116011, China

## Abstract

Abnormality of glutathione peroxidase (GPx) is involved in the etiology and pathogenesis of vitiligo. However, the results were controversial.* Aim*. The purpose of this meta-analysis is to compare the levels of GPx between vitiligo patients and healthy controls.* Methods.* Relevant published articles were searched according to eligibility criteria. A meta-analysis was conducted to pool estimates of the standardized mean difference (SMD) with 95% confidence interval (CI).* Results.* Twenty-three studies with a total of 1076 vitiligo patients and 770 healthy controls were included. The pooled meta-analysis showed that patients with vitiligo had equivalent levels of GPx with the healthy controls (SMD = −0.47, 95% CI: −1.03 to 0.08, and *p* = 0.095). Further subgroup analysis showed that the GPx levels of Asian patients or segmental vitiligo patients were, respectively, lower than those of healthy controls (Asian: SMD = −0.47, 95% CI: −1.08 to 0.14, and *p* = 0.001; segmental: SMD = −3.59, 95% CI: −6.38 to −0.80, and *p* = 0.012). Furthermore, the GPx levels in serum/plasma were significantly decreased in either stable or active vitiligo patients, comparing to healthy controls (stable: SMD = −2.01, 95% CI: −3.52 to −0.49, and *p* = 0.009; active: SMD = −2.34, 95% CI: −4.07 to −0.61, and *p* = 0.008).* Conclusion*. This meta-analysis showed a significant association between low GPx level and vitiligo.

## 1. Introduction

Vitiligo is an idiopathic, acquired pigmentation disorder of skin and/or mucosa, with clinical manifestations of porcelain white patches. It is considered to be a multifactorial and polygenic disease caused by the destruction of melanocytes [1]. Amongst others, oxidative stress is considered to be one of the causative factors in the pathogenesis of vitiligo [2].

Glutathione peroxidase (GPx) is the general name of an enzyme family with peroxidase activity. It protects cells from oxidative damage through decreasing lipid hydroperoxides to their corresponding alcohols or reducing free hydrogen peroxide to water [3]. In vitiligo, many researches about this antioxidant marker have been sought, but the conclusions were conflicting. Some researchers reported elevated level, whereas others showed no change or reduced level. Due to the inconsistent results, we do the meta-analysis to clarify the GPx level in vitiligo patients.

## 2. Materials and Methods

### 2.1. Search Strategy

The PubMed, Cochrane Library, Web of Science, Chinese National Knowledge Infrastructure (CNKI), and Wan Fang Med Online were searched by two independent investigators using the search terms (“vitiligo”) and (“glutathione peroxidase” or “GPx” or “GSH-Px” or “oxidant” or “antioxidant”). Additional potential relevant articles were further retrieved through a manual search of references from original reports. The research dated from the earliest time to December 2015.

### 2.2. Eligibility Criteria and Excluded Studies

We sought existing studies published in English or Chinese. Articles were included in this meta-analysis if (1) the case group consisted of vitiligo patients and the control group included healthy individuals and (2) the outcome measures reported quantitative GPx level (mean ± standard deviation). After reading the title and abstract, we excluded a study if it (1) was an animal or in vitro experiment, (2) was a case report or a review, and (3) consisted of duplicate data with other study. All studies were deliberately reviewed by two investigators to decide whether to be included.

### 2.3. Data Extraction

Two investigators independently screened studies for eligible articles. The following items including the first author, year of publication, nation, sample size, sources, test method of GPx, GPx estimated value, unit, type, and stage of vitiligo were extracted. If there were discrepancies, they would reach a consensus through discussion and reexamination or seeking help to a third investigator.

### 2.4. Quality Assessment

To estimate the quality of included studies, the Newcastle-Ottawa Scale (NOS) criteria were used by two investigators independently [4]. The NOS criteria were scored based on three aspects: (1) subject selection, 0~4, (2) comparability of subject, 0~2, and (3) clinical outcome, 0~3. Total NOS scores range from 0 (the lowest) to 9 (the highest). Any discrepancy between the two investigators on NOS scores of the enrolled studies was resolved by discussion or consultation with a third investigator.

### 2.5. Statistical Analysis

The standard mean difference (SMD) for the effect and corresponding 95% CIs were calculated from the original data of the appropriate studies in fixed effects model (Mantel-Haenszel method) or random-effects model (DerSimonian and Laird method). The random-effects model was applied when heterogeneity existed among studies, while the fixed effects model was applied when there was no statistical heterogeneity. In order to test for comparability, heterogeneity across the included studies was evaluated by Cochran *Q* test and *I* ^2^ test [5]. Subgroup meta-analyses were conducted according to race (Caucasian versus Asian), stage (active or stable), type (segmental or nonsegmental), or source of sample. The funnel plot was constructed to assess the effect of publication bias on the validity of the estimates. The symmetry of the funnel plot was further evaluated by Egger's linear regression test [6]. All tests were two-sided, and a *p*value of < 0.05 was regarded as statistically significant. Stata version 11.0 software (StataCorp., College Station, TX, USA) was performed for statistical analysis.

## 3. Results

### 3.1. Eligible Studies

**Figure 1:**
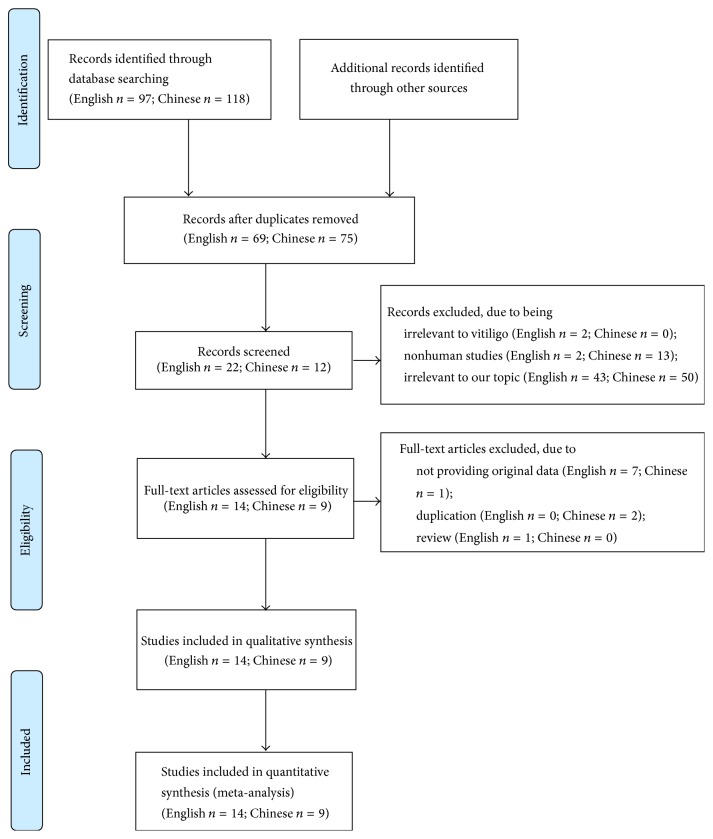
Flow diagram of screened and included studies.

We identified 215 studies according to search strategy as shown in Figure 1. After carefully reviewing and screening, 23 articles [3, 7–28] were finally included in the meta-analysis. The characteristic and methodological qualities of these studies were shown in Table 1. The overall study quality ranged from 5 to 7 stars. Of the 23 studies, the sample resource of 20 studies was either serum, plasma, erythrocyte, blood, skin, or blister fluid, and other 3 studies, respectively, tested the GPx level in two sample sources. So the total number of comparisons used in the meta-analysis was 26. The race of all included studies was Caucasian or Asian population. The levels of GPx stratified by sample sources and races were listed in Table 2.

### 3.2. The Levels of GPx in Vitiligo Patients and Healthy Controls

**Figure 2:**
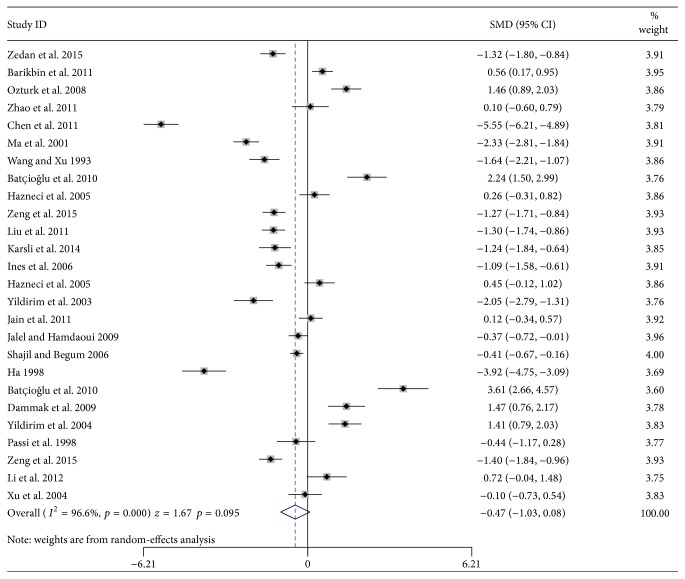
Forest plots of studies in glutathione peroxidase levels for subjects with vitiligo patients versus healthy controls.

Random-effects model was applied to the pooled meta-analysis, as statistical heterogeneity existed among studies (*χ* ^2^ = 741.66, *p* = 0.000, and *I* ^2^ = 96.6%). The results indicated that patients with vitiligo had equivalent levels of GPx with the healthy controls (SMD = −0.47, 95% CI: −1.03 to 0.08, and *p* = 0.095) (Figure 2).

**Figure 3:**
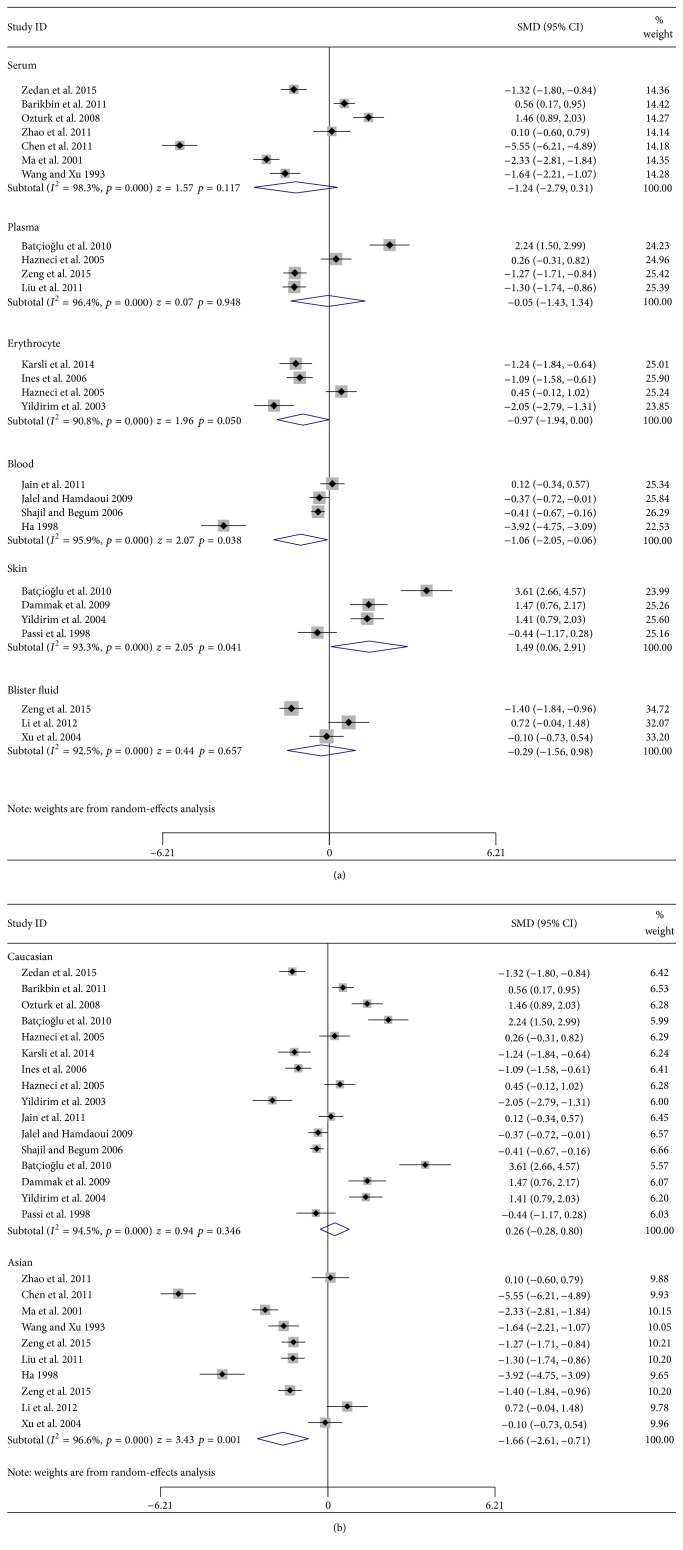
Subgroup analyses of studies in glutathione peroxidase levels for subjects with vitiligo versus healthy controls stratified by (a) sample sources and (b) races.

**Figure 4:**
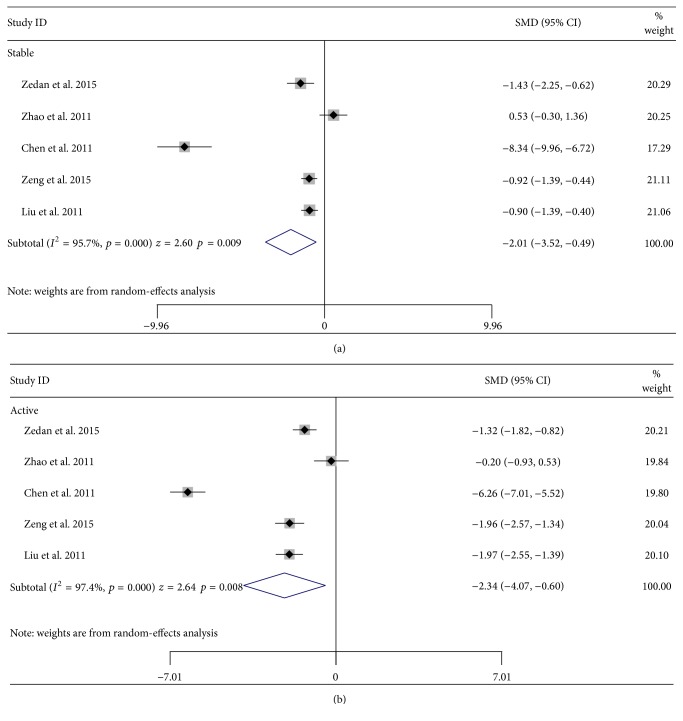
Subgroup analyses of studies in glutathione peroxidase levels in serum/plasma stratified by vitiligo stage. (a) Vitiligo at stable stage versus healthy controls and (b) vitiligo at active stage versus healthy controls.

Further subgroup analysis stratified by sample sources indicated that vitiligo patients had higher GPx levels than controls in skin (SMD = 1.49, 95% CI: 0.06 to 2.91, and *p* = 0.041) and lower GPx levels than controls in blood (SMD = −1.06, 95% CI: −2.06 to −0.06, and *p* = 0.038). No difference was seen in the source of serum (SMD = −1.24, 95% CI: −2.79 to 0.31, and *p* = 0.117), plasma (SMD = −0.05, 95% CI: −1.43 to 1.34, and *p* = 0.948), erythrocyte (SMD = −0.97, 95% CI: −1.94 to 0.00, and *p* = 0.050), or blister fluid (SMD = −0.29, 95% CI: −1.56 to 0.98, and *p* = 0.657) (Figure 3(a)). The analysis stratified by race indicated that vitiligo patients in Asian populations had lower GPx levels than controls (SMD = −0.47, 95% CI: −1.08 to 0.14, and *p* = 0.001), but no difference was shown in Caucasian populations (SMD = 0.259, 95% CI: −0.28 to 0.80, and *p* = 0.346) (Figure 3(b)). Five articles were included in the subgroup analyses stratified by stage and sample source of serum/plasma (Table 3). The results indicated that the vitiligo patients at either stable stage or active stage had lower GPx levels in serum/plasma compared to controls (stable: SMD = −2.01, 95% CI: −3.52 to −0.49, and *p* = 0.009; active: SMD = −2.34, 95% CI: −4.07 to −0.61, and *p* = 0.008) (Figures 4(a) and 4(b)). No significant difference was observed between stable stage and active stage (SMD = 0.50, 95% CI: −0.02 to 1.01, and *p* = 0.058). Three articles were included in the subgroup analyses stratified by vitiligo type (Table 4). Segmental vitiligo patients had lower GPx levels compared to controls (SMD = −3.59, 95% CI: −6.38 to −0.80, and *p* = 0.012). No significant difference was observed between nonsegmental vitiligo patients and controls (SMD = −2.81, 95% CI: −5.71 to 0.10, and *p* = 0.058) or between segmental and nonsegmental vitiligo patients (SMD = −0.18, 95% CI: −0.47 to 0.11, and *p* = 0.230).

### 3.3. Metaregression and Sensitivity Analyses

**Figure 5:**
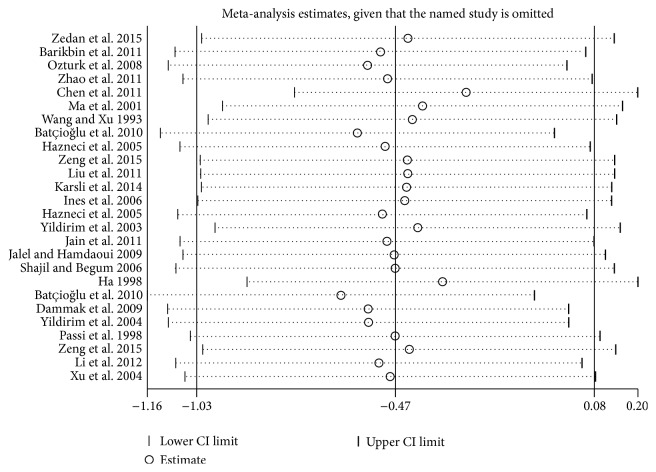
Forest plots for the sensitive analysis.

Univariate and multivariate metaregression analyses were used to explore possible sources of heterogeneity. The results showed that race could be the major source of heterogeneity (Table 5). The results of sensitivity analysis suggested that no individual studies significantly affected the pooled results, indicating a statistically robust result (Figure 5).

### 3.4. Publication Bias

We used Egger's test to estimate the possibility of publication bias. The results showed that there was no obvious evidence of publication bias (*t* = 0.32, *p* = 0.754).

## 4. Discussion

Oxidative stress inducing vitiligo is based on the fact that some intermediates such as 3,4-dihydroxyphenylalanine (dopa), dopachrome, and 5,6-dihydroxyindole are created during melanin biosynthesis [29]. The final result of these changes results in the continuous increase of hydrogen peroxide (H_2_O_2_), which restrains the antioxidative enzyme activity leading to the destruction of melanocytes [30]. Therefore, antioxidants are important to nullify the harmful radical-mediated reactions. GPx is a group of antioxidative markers against free radicals by detoxification and has been considered to be involved in the pathogenesis of many skin diseases [31–33]. Our meta-analysis investigated whether GPx is involved in the development of vitiligo. The results of included articles involving 26 comparisons on the relationship of GPx and vitiligo were controversial; that is, respective 50%, 31%, and 19% comparisons showed lower, equal, and higher levels in vitiligo samples. The difference may relate to the variations in the population race, disease type, activity, duration, sample sources, or detection method.

Till now, no meta-analysis has reported the association between the GPx level and vitiligo. The pooled meta-analysis results of all the comparisons indicated that the GPx levels in vitiligo patients were similar to healthy controls. As statistical heterogeneity existed among studies, we did further subgroup analysis. The results indicated a significant relationship between low GPx level and vitiligo incidence.

Our subgroup analysis showed that Asian vitiligo patients showed lower levels of GPx than the controls, but no difference was shown between Caucasian populations and healthy controls. The metaregression results, which showed that race could be the major source of heterogeneity of pooled meta-analysis, supported the above subgroup analysis results. The majority of previous studies have used serum or plasma to measure oxidant or antioxidant levels. In the present meta-analysis, whatever stable vitiligo patients or active vitiligo patients had lower serum/plasma levels of GPx than the controls. The patients with segmental type also had decreased GPx levels comparing to healthy controls. These results suggested that low GPx level may contribute to the pathogenesis of vitiligo in Asian population, unlike Caucasian population. The low level in serum/plasma was associated with vitiligo incidence, at whatever active stage or stable stage, especially in segmental vitiligo. Oxidative stress induced accumulation of toxic-free radicals may have a pathophysiologic role in the initiation and progression of vitiligo [2]. Reactive oxygen species (ROS) are scavenged by antioxidant defence mechanisms. Depletion of the endogenous antioxidants including GPx can overwhelm antioxidant defence mechanisms, resulting in oxidative stress medicated vitiligo. Besides, allelic variants in GPx gene may be associated with low levels of GPx activity [34, 35]. One previous study indicated that GPx polymorphism may contribute to the reduced GPx activity and the prevalence of vitiligo in Gujarat population [36].

In conclusion, this meta-analysis showed a significant association between low GPx level and vitiligo for Asian population or segmental patients. The low level in serum/plasma was associated with vitiligo incidence, at whatever active or stable stage. Nonetheless, the conclusion could not be completely confirmed as there are some limitations. The limited number, small sample sizes of studies, and methodological diversities may weaken the statistical power. More large-sample studies of higher quality should be done to verify the conclusions.

## Figures and Tables

**Table 1:** Characteristic and methodological qualities of included studies.

Study	Nation	Number of participants (patients/controls)	Source	Test method	Unit	Type	Stage	NOS score
Zedan et al. [3]	Egypt	60/30	Serum	1	U/L	Generalized, localized	Stable, active	7
Barikbin et al. [7]	Iran	60/45	Serum	2	U/L	Vulgaris	Active	5
Ozturk et al. [8]	Turkey	30/30	Serum	4	U/mg protein	Generalized	Stable	5
Zhao et al. [9]	China	40/10	Serum	—	—	—	Stable, active	5
Chen et al. [10]	China	132/50	Serum	3	U/L	Vulgaris, segmental	Stable, active	5
Ma et al. [11]	China	69/44	Serum	3	U/L	Vulgaris, segmental	—	5
Wang and Xu [12]	China	34/30	Serum	1	U/mg protein	—	—	7
Batçioğlu et al. [13]	Turkey	37/15	Plasma	4	U/mg protein	—	—	7
Hazneci et al. [14]	Turkey	23/25	Plasma	5	U/mg protein	Vulgaris	Active	6
Zeng et al. [15]	China	50/50	Plasma	—	U/L	—	Stable, active	5
Liu et al. [16]	China	60/40	Plasma	3	U	—	Stable, active	7
Karsli et al. [17]	Turkey	24/27	Erythrocyte	1	U/g Hb	Generalized	—	7
Ines et al. [18]	Tunisia	36/40	Erythrocyte	6	U/g protein	—	Stable, active	5
Hazneci et al. [14]	Turkey	23/25	Erythrocyte	1	U/g Hb	Vulgaris	Active	6
Yildirim et al. [19]	Turkey	24/20	Erythrocyte	1	U/g Hb	Generalized	Stable	5
Jain et al. [20]	India	75/25	Blood	1	U/L	—	Stable, active	5
Jalel and Hamdaoui [21]	Tunisia	60/62	Blood	1	U/L	—	—	5
Shajil and Begum [22]	India	124/126	Blood	7	U/mg protein	Segmental, nonsegmental	—	5
Ha [23]	China	35/31	Blood	—	U	—	Active	5
Batçioğlu et al. [13]	Turkey	33/15	Skin	4	U/mg protein	—	—	7
Dammak et al. [24]	Tunisia	20/20	Skin	1	U/mg protein	Vulgaris	Stable, active	7
Yildirim et al. [25]	Turkey	25/25	Skin	1	U/mg protein	Generalized	Stable	5
Passi et al. [26]	Italy	15/15	Skin	1	U/mg protein	—	Active	7
Zeng et al. [15]	China	50/50	Blister fluid	—	U/L	—	Stable, active	5
Li et al. [27]	China	24/10	Blister fluid	3	U	—	Stable, active	7
Xu et al. [28]	China	19 (self control)	Blister fluid	3	U	—	Stable	7

*Note*. NOS, Newcastle-Ottawa Scale; 1: Paglia and Valentine's method (1967); 2: flameless atomic absorption (graphite furnace) method; 3: DTNB colorimetry; 4: Lawrence and Burk's method (1976); 5: Najwa's method (1990); 6: Paglia's method (2002); 7: Beutler's method (1989).

**Table 2:** The level of glutathione peroxidase of vitiligo patients stratified by sample sources and races (mean ± SD).

Study	Race	Unit	Case	Control	Source
Zedan et al. [3]	Caucasian	U/L	0.29 ± 0.14	0.47 ± 0.13	Serum
Barikbin et al. [7]	Caucasian	U/L	191.300 ± 14.95	183.288 ± 13.42	Serum
Ozturk et al. [8]	Caucasian	U/mg protein	0.550 ± 0.077	0.439 ± 0.075	Serum
Zhao et al. [9]	Asian	—	292.21 ± 127.42	280.88 ± 47.25	Serum
Chen et al. [10]	Asian	U/L	140030 ± 15260	216440 ± 8610	Serum
Ma et al. [11]	Asian	U/L	102.08 ± 19.32	154.76 ± 27.06	Serum
Wang and Xu [12]	Asian	U/mg protein	0.000981 ± 0.000257	0.001722 ± 0.000602	Serum
Batçioğlu et al. [13]	Caucasian	U/mg protein	381.57 ± 12.67	346.13 ± 21.90	Plasma
Hazneci et al. [14]	Caucasian	U/mg protein	0.205 ± 0.192	0.171 ± 0.012	Plasma
Zeng et al. [15]	Asian	U/L	98.44 ± 14.23	114.33 ± 10.41	Plasma
Liu et al. [16]	Asian	U	96.40 ± 15.78	115.14 ± 12.20	Plasma
Karsli et al. [17]	Caucasian	U/g Hb	13.71 ± 3.85	18.87 ± 4.42	Erythrocyte
Ines et al. [18]	Caucasian	U/g protein	1160 ± 440	1890 ± 820	Erythrocyte
Hazneci et al. [14]	Caucasian	U/g Hb	97836.86 ± 17947.61	90257.83 ± 15776.65	Erythrocyte
Yildirim et al. [19]	Caucasian	U/g Hb	31.34 ± 14.3	101.57 ± 48.4	Erythrocyte
Jain et al. [20]	Caucasian	U/L	4004 ± 482.34	3945 ± 552	Blood
Jalel and Hamdaoui [21]	Caucasian	U/L	42 ± 23.24	49 ± 14.17	Blood
Shajil and Begum [22]	Caucasian	U/mg protein	944.55 ± 160.92	1036.8 ± 269.6	Blood
Ha [23]	Asian	U	128.18 ± 18.35	206.14 ± 21.50	Blood
Batçioğlu et al. [13]	Caucasian	U/mg protein	170.98 ± 12.35	121.91 ± 16.03	Skin
Dammak et al. [24]	Caucasian	U/mg protein	2.73 ± 0.81	1.78 ± 0.43	Skin
Yildirim et al. [25]	Caucasian	U/mg protein	3.72 ± 2.09	1.58 ± 0.48	Skin
Passi et al. [26]	Caucasian	U/mg protein	0.43 ± 0.10	0.47 ± 0.08	Skin
Zeng et al. [15]	Asian	U/L	86.53 ± 7.83	98.26 ± 8.87	Blister fluid
Li et al. [27]	Asian	U	148.73 ± 51.91	115.01 ± 29.57	Blister fluid
Xu et al. [28]	Asian	U	90.67 ± 63.07	96.76 ± 63.51	Blister fluid

**Table 3:** The level of glutathione peroxidase of vitiligo patients stratified by stage and healthy controls (mean ± SD).

Study	Unit	Case		Control	Source
		Stable	Active		
Zedan et al. [3]	U/L	0.27 ± 0.17 (*n* = 9)	0.29 ± 0.14 (*n* = 48)	0.47 ± 0.13 (*n* = 30)	Serum
Zhao et al. [9]	—	346.93 ± 156.84 (*n* = 14)	262.75 ± 103.54 (*n* = 26)	280.88 ± 47.25 (*n* = 10)	Serum
Chen et al. [10]	U/L	139120 ± 11760 (*n* = 12)	140940 ± 13210 (*n* = 120)	216440 ± 8610 (*n* = 50)	Serum
Zeng et al. [15]	U/L	104.39 ± 11.49 (*n* = 30)	88.74 ± 18.23 (*n* = 20)	114.33 ± 10.41 (*n* = 50)	Plasma
Liu et al. [16]	U	103.08 ± 14.95 (*n* = 30)	89.72 ± 13.80 (*n* = 30)	115.14 ± 12.20 (*n* = 40)	Plasma
Ines et al. [18]	U/g protein	1250 ± 470 (*n* = 18)	1060 ± 380 (*n* = 18)	1890 ± 820 (*n* = 40)	Erythrocyte
Jain et al. [20]	U/L	3990 ± 459 (*n* = 25)	4011 ± 498 (*n* = 50)	3945 ± 552 (*n* = 25)	Blood
Ha [23]	U/mg protein	2.48 ± 0.81 (*n* = 10)	2.98 ± 0.77 (*n* = 10)	1.78 ± 0.43 (*n* = 20)	Skin
Zeng et al. [15]	U/L	95.33 ± 9.37 (*n* = 30)	80.69 ± 10.38 (*n* = 20)	98.26 ± 8.87 (*n* = 50)	Blister fluid
Passi et al. [26]	U	92.32 ± 23.67 (*n* = 9)	182.58 ± 73.67 (*n* = 15)	115.01 ± 29.57 (*n* = 10)	Blister fluid

**Table 4:** The level of glutathione peroxidase of segmental vitiligo patients, nonsegmental vitiligo patients, and healthy controls (mean ± SD).

Study	Unit	Case		Control	Source
		Segmental	Nonsegmental		
Chen et al. [10]	U/L	141500 ± 9210 (*n* = 8)	138560 ± 14620 (*n* = 124)	216440 ± 8610 (*n* = 50)	Serum
Ma et al. [11]	U/L	98.33 ± 20.34 (*n* = 23)	103.95 ± 18.73 (*n* = 46)	154.76 ± 27.06 (*n* = 44)	Serum
Shajil and Begum [22]	U/mg protein	916.8 ± 183.3 (*n* = 30)	953.4 ± 151.9 (*n* = 94)	1036.8 ± 269.6 (*n* = 126)	Blood

**Table 5:** Metaregression analyses of potential source of heterogeneity.

Heterogeneity factors	Coefficient	Std. Err.	*z*	*p* > |*z*|	95% CI	
					LL	UL
Race						
Univariate	−1.940708	.6522389	−2.98	0.003	−3.219072	−.6623431
Multivariate	−1.811697	.6340008	−2.86	0.004	−3.054315	−.569078
Country						
Univariate	−.158666	.2952626	−0.54	0.591	−.73737	.420038
Multivariate	−.3211	.27018	−1.19	0.235	−.8506431	.2084431
Sample Source						
Univariate	.3021601	.2049701	1.47	0.140	−.099574	.7038942
Multivariate	.370906	.1947404	1.90	0.057	−.0107781	.7525901

*Note*. Std. Err., standard error; 95% CI, 95% confidence interval; UL, upper limit; LL, lower limit.
