# Corrigendum to “Glutathione Peroxidase Level in Patients with Vitiligo: A Meta-Analysis”

**DOI:** 10.1155/2017/6707162

**Published:** 2017-07-12

**Authors:** Bi-huan Xiao, Meihui Shi, Hongqiang Chen, Shaoshan Cui, Xing-Hua Gao, Yan Wu, Hong-Duo Chen

**Affiliations:** ^1^Department of Dermatology, No. 1 Hospital of China Medical University, Shenyang 110001, China; ^2^Department of General Surgery, Shandong Qianfoshan Hospital, Jinan 250014, China; ^3^Department of Dermatology, The First Affiliated Hospital of Dalian Medical University, Dalian 116011, China

In the article titled “Glutathione Peroxidase Level in Patients with Vitiligo: A Meta-Analysis” [1], the order of the authors in the author and correspondence list was incorrect. The revised author and correspondence lists are shown above.
